# Using system dynamics modelling to estimate the costs of relaxing health system constraints: a case study of tuberculosis prevention and control interventions in South Africa

**DOI:** 10.1093/heapol/czab155

**Published:** 2021-12-24

**Authors:** Fiammetta M Bozzani, Karin Diaconu, Gabriela B Gomez, Aaron S Karat, Karina Kielmann, Alison D Grant, Anna Vassall

**Affiliations:** Department of Global Health and Development, London School of Hygiene & Tropical Medicine, 15-17 Tavistock Place, London WC1H 9SH, UK; Institute for Global Health and Development, Queen Margaret University, Queen Margaret University Way, Musselburgh EH21 6UU, UK; Department of Global Health and Development, London School of Hygiene & Tropical Medicine, 15-17 Tavistock Place, London WC1H 9SH, UK; Institute for Global Health and Development, Queen Margaret University, Queen Margaret University Way, Musselburgh EH21 6UU, UK; TB Centre, London School of Hygiene & Tropical Medicine, Keppel Street, London WC1E 7HT, UK; Institute for Global Health and Development, Queen Margaret University, Queen Margaret University Way, Musselburgh EH21 6UU, UK; TB Centre, London School of Hygiene & Tropical Medicine, Keppel Street, London WC1E 7HT, UK; Africa Health Research Institute, School of Laboratory Medicine & Medical Sciences, College of Health Sciences, University of KwaZulu-Natal, Nelson R. Mandela Medical School, 719 Umbilo Road, Umbilo, Durban 4001, South Africa; School of Public Health, University of the Witwatersrand, 27 Street, Andrews Road, Parktown 2193, South Africa; Department of Global Health and Development, London School of Hygiene & Tropical Medicine, 15-17 Tavistock Place, London WC1H 9SH, UK

**Keywords:** Infectious disease control, tuberculosis, system dynamics modelling, economic evaluation

## Abstract

Health system constraints are increasingly recognized as an important addition to model-based analyses of disease control interventions, as they affect achievable impact and scale. Enabling activities implemented alongside interventions to relax constraints and reach the intended coverage may incur additional costs, which should be considered in priority setting decisions. We explore the use of group model building, a participatory system dynamics modelling technique, for eliciting information from key stakeholders on the constraints that apply to tuberculosis infection prevention and control processes within primary healthcare clinics in South Africa. This information was used to design feasible interventions, including the necessary enablers to relax existing constraints. Intervention and enabler costs were then calculated at two clinics in KwaZulu-Natal using input prices and quantities from the published literature and local suppliers. Among the proposed interventions, the most inexpensive was retrofitting buildings to improve ventilation (US$1644 per year), followed by maximizing the use of community sites for medication collection among stable patients on antiretroviral therapy (ART; US$3753) and introducing appointments systems to reduce crowding (US$9302). Enablers identified included enhanced staff training, supervision and patient engagement activities to support behaviour change and local ownership. Several of the enablers identified by the stakeholders, such as obtaining building permissions or improving information flow between levels of the health systems, were not amenable to costing. Despite this limitation, an approach to costing rooted in system dynamics modelling can be successfully applied in economic evaluations to more accurately estimate the ‘real world’ opportunity cost of intervention options. Further empirical research applying this approach to different intervention types (e.g. new preventive technologies or diagnostics) may identify interventions that are not cost-effective in specific contexts based on the size of the required investment in enablers.

**Key messages**

- Estimating the full costs of health system investments to successfully implement tuberculosis infection prevention and control interventions in South African clinics is essential for priority setting
- Group model building, a participatory system dynamics modelling (SDM) technique, may assist with designing feasible interventions and identifying activities that can be costed to overcome health system constraints and achieve implementation targets
- Interventions requiring large upfront capital investments, such as retrofitting buildings to improve ventilations or installing UV germicidal irradiation systems, are cheaper over time than interventions requiring behaviour change (e.g. ensuring windows and doors are kept open) due to the higher relative share of enabling costs (e.g. of intensified training and supervision) required to make them sustainable
- SDM-informed costing allows for a comprehensive view of the health system influences on intervention impact and feasibility, in a way that may be superior to other, less participatory stakeholder consultation methods

## Introduction

Reducing the transmission of *Mycobacterium tuberculosis* (*Mtb*) in primary care clinics and other health care settings is a priority on tuberculosis (TB) infection prevention and control (IPC) agenda in South Africa. Transmission of drug-resistant (DR) *Mtb* is documented within health facilities (O’Donnell *et al.*, 2010; World Health Organization, 2019). Moreover, recent mathematical modelling evidence generated using data from KwaZulu-Natal implies that the risk of *Mtb* transmission in clinics in high human immunodeficiency virus (HIV) burden settings may be higher than contact data would suggest for both health care workers and patients (Mccreesh *et al.*, 2020). Guidelines for airborne IPC in health facilities are widely available (National Department of Health, 2015; World Health Organization, 2019), but numerous implementation challenges are documented (Barrera-Cancedda *et al.*, 2019). These are linked to a range of contributing factors including clinic design, climatic conditions, work practices and the organization of care, risk perceptions, competing priorities, organizational culture and concerns about stigma (Claassens *et al.*, 2013; Farley *et al.*, 2012; WHO, 2014). This is defined as an interdisciplinary approach that (1) contextualizes clinic-level TB IPC processes within the structure of the broader health system; and (2) analyses interactions across health system components (Kielmann *et al.*, 2020).

Mathematical models of disease transmission are increasingly recognized as a vital tool for understanding health system functioning and optimization, given their capability to simulate the behaviour of complex adaptive systems (Cassidy *et al.*, 2019). Mathematical models allow for the use of locally relevant epidemiological parameters, and model outputs can be combined with local unit costs (or cost functions). In this way, models enable analysts to explore the efficiency of investments in infection control in specific settings and can assist with priority setting and resource allocation at the country level. Most recently, studies have begun exploring possibilities for parameterizing models with data on the health system constraints affecting real-world intervention implementation (Bozzani *et al.*, 2021; Vassall *et al.*, 2016). Constraints can operate through elements of the health system’s ‘hardware’, e.g. in the form of physical inputs shortages (human resources, diagnostic equipment and consumables, drugs), or through its ‘software’, as factors influencing the decision-making process (such as equity and other political and social considerations) (Sheikh *et al.*, 2011). Both types of constraints might impact *feasibility*, through the pace of scale-up, and *effectiveness* of interventions, by reducing achievable coverage. Their impact can be particularly severe in low- and middle-income countries, where budgets are limited and new interventions often represent a large proportion of the available funds (Mills, 2014). Displacing resources can thus have a substantial health impact and, for this reason, it is vital to produce estimates of the value of these resources (opportunity cost) that is accurate and complete, including the costs of any additional activities alongside intervention implementation (‘enablers’) that may be necessary to overcome the constraints.

While it is possible to use routine cost data for this purpose, building cost parameters that account for the additional expenses incurred to relieve the constraints and achieve the intended intervention targets in a ‘real-world’ setting poses novel difficulties for analysts (Bozzani *et al.*, 2018). In particular, there is no consensus currently on the best way to elicit comprehensive information on the constraints that apply to a specific setting and intervention (i.e. on the dynamic interactions between the intervention and specific elements of the health system) and their impact on successful implementation and scale-up. In this paper, we use TB IPC interventions as a case study to illustrate how system dynamics modelling (SDM) techniques can be used to take a whole systems approach to costing, that includes information on health system constraints and on the actions required to relax them at different levels of the health system.

## Materials and methods

Ethics approval for the study was granted by the research ethics committees of the authors’ institutes.

### Study setting

The costing exercise presented in this case study was undertaken at two clinics in rural KwaZulu-Natal, South Africa, as part of *Umoya omuhle*, a multidisciplinary project aimed at understanding the drivers of nosocomial transmission of *Mtb* in primary healthcare facilities (Kielmann *et al.*, 2020). *Umoya omuhle* collected a wealth of information on the policies, norms and values governing TB-IPC processes for clinic staff and patients, as well as on the infrastructure and resources for TB-IPC, implementation challenges, and on existing levels of indoor ventilation and congregation to parametrize a model of *Mtb* transmission in the clinics and surrounding communities (Colvin *et al.*, 2020; Mccreesh *et al.*, 2020; Voce, 2020).

### System dynamics modelling

The TB IPC interventions investigated in the *Umoya omuhle* study were identified and designed using an SDM approach described in detail elsewhere (Diaconu *et al.*, 2021). Briefly, SDM is a complexity science method increasingly applied in health policy and systems research (Chang *et al.*, 2017; Darabi and Hosseinichimeh, 2020). The approach was selected due to its focus on health systems as complex adaptive systems, which allows for the translation of this complexity in intervention design (Paina and Peters, 2012). Of particular value for this costing exercise is the fact that SDM can produce a model of the health system that acknowledges and explicitly considers the dynamic interaction between interventions and the underlying health system, highlighting where constraints can arise and additional costs may be incurred to address these constraints (Verguet *et al.*, 2019).

Group model building, a participatory method used for qualitative SDM elaboration of causal loop diagrams, was used in *Umoya omuhle* to learn about the feedback loops and non-linear effects that are present in the TB IPC system in South Africa and that might cause unexpected or unintended outcomes in response to interventions and policy changes (Iwelunmor *et al.*, 2015; Northridge and Metcalf, 2016). The group model-building exercise consisted of two one-day workshops, the first with national- and provincial-level policymakers and the second with the district- and facility-level health professionals, patient advocates and public health practitioners in a range of specialties, including managers, researchers and architects. During the workshops, participants were guided to develop causal loop diagrams which represented their understanding of the current dynamics shaping nosocomial *Mtb* transmission at the clinic level, including points of fragility within the TB IPC system and, among those, leverage points where interventions would be feasible. More detail on the elicitation methods and the causal loop diagram summarising the dynamics at play are presented in Supplementary File S1.

This information then fed into the design of interventions that would be effective at reducing nosocomial *Mtb* transmission and that would take existing constraints into account, incorporating to the extent feasible the necessary enablers to overcome these constraints. Interventions were thus conceptualized as including a set of core activities, necessary for delivering the intervention in any setting (e.g. the staff time to open a window), and a set of enabler activities identified during the group model building as necessary to ensure interventions are feasible in South African clinics (e.g. increased training and district-level supervision). Pathways of action of the identified interventions and enablers were described through a process of iterative review and revision of the causal loop diagrams and free lists generated by stakeholders during the workshops, integrated with the qualitative evidence gathered by the wider *Umoya Omuhle* project.

### Intervention costing

Unit costs for core intervention activities and enablers were estimated using price and quantity data from the published literature and quotes from local suppliers. Unit costs captured the incremental economic costs of all core activities, including the opportunity cost of staff time, recognizing that even activities that are not time-consuming and that are already implemented to some degree, such as opening windows and doors to improve ventilation or directing queuing patients, will need dedicated staff to increase their feasibility and impact compared to current levels (Islam *et al.*, 2021). Quantity assumptions were supplemented with data from interviews with facility managers, IPC managers and nurses at the *Umoya omuhle* study facilities, who were asked about input requirements, including staff time, for carrying out hypothetical tasks. Capital investments and other start-up costs were annualized using a 3% discount rate for future costs. All costs are presented in 2019 US$.

All core activities and enablers that emerged from the group model building sessions as desirable to improve the feasibility and impact of the proposed interventions were considered for inclusion in the cost model. However, reliable data sources could not be identified for some of the proposed activities that were entirely novel (e.g. electronic health records linkage to appointment systems), above service-level (e.g. redesigning training materials using routine monitoring and evaluation data) or outside the remit of the Department of Health (e.g. improving transport links to clinics to ensure the viability of appointment systems). Other activities were acknowledged as central to the intervention but excluded from the costing exercise, as they did not represent an actual cost, i.e. they referred to barriers that could not be overcome through financial investments (e.g. having to obtain permission from the district to carry out clinic building modifications).

The final list of interventions included in the cost model is presented in Table 1, which details the core activities and enablers costed as well as those enablers indicated as desirable by the SDM participants that could not be costed. A full list of price and quantity assumptions is presented in Supplementary File S1. Unit costs and underlying assumptions were checked and validated with SDM participants multiple times during model development, first through monthly drop-in virtual meetings and finally during a second SDM workshop, where preliminary costing results were presented to participants. Their feedback was then incorporated into the analysis to produce the final estimates.

**Table 1.** Description of interventions costed

Intervention	Core activities modelled	Enablers modelled	Enabler as % of unit cost	Enablers not modelled
1: Improving ventilation by opening doors and windows	One clinical staff doing a round of the clinic every hour	One-day training for all clinical staff every 3 years and intensified supervision from the district. Electric heaters/fans to ensure thermal comfort.	23%	Other communication materials and training formats re-designed based on M&E data
2: Building retrofits	Raising roof of waiting area, installing turbine ventilators and lattice brickwork	None	0%	Obtaining permissions from district, community workshop to decide which retrofits
3: UVGI	UV lights installation, maintenance, calibration and electricity	One-day training for all clinical staff every 3 years	11%	National level processes for lifting existing moratorium and launching new tender
4: Surgical mask wearing for patients and N95 respirators for staff	One N95 respirator per staff every five shifts, fitted annually (50% coverage). One surgical mask per patient per visit (70% coverage)	One-day training for all clinical staff every 3 years. Free leaflet for one in ten patients disseminated around clinic	25%	Other communication materials, training formats or community events redesigned based on M&E data
5: Maximizing use of existing CCMDD facilities	None	Half-day training for staff involved in implementation every three years. Once-off community workshops	100%	Providing additional CCMMD pick-up points outside of clinics, particularly where no private pharmacies available within catchment area
6: Queue management system	One nurse triaging patients and one lay staff directing queues	Half-day training for staff involved in implementation every 3 years and intensified supervision from district. Once-off community workshops. Covered outdoor waiting area	46%	Other ways of addressing ‘queue anxiety’ such as numbered tickets, re-designing training formats and materials incorporating M&E data
7: Appointments system	1 hour per day for clerk to pre-retrieve files and record appointments. 1 hour for public awareness messaging in waiting area	Half-day training for staff involved in implementation every 3 years. Once-off community workshops.	54%	Addressing issues with transportation availability throughout the day, redesigning training formats and materials incorporating M&E data

M&E: Monitoring and Evaluation.

## Results

**Figure 1.**
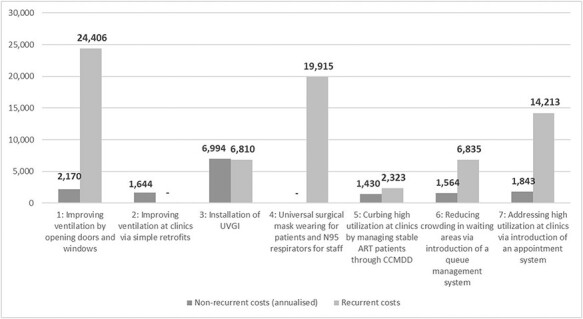
Incremental annual costs of interventions and enabler activities at two clinics in KwaZulu-Natal, 2019 US$

Incremental annual costs of each intervention option, including upfront capital investment and recurrent costs of all intervention activities and enablers, are reported in Figure 1. The least expensive interventions considered were the retrofitting of buildings to improve ventilation, which consisted of relatively cheap and long-lasting building modifications such as installing turbine ventilators, substituting portions of walls and windows with lattice brickwork and raising waiting area roofs; and expanding the decentralized treatment management of stable ART patients through the Central Chronic Medicines Dispensing and Distribution (CCMDD) system (Dorward *et al.*, 2020). The switch from more frequent monitoring at ART clinics to 6-monthly repeat prescriptions and drug dispensing through CCMDD might ultimately be cost-saving for the health system, as it promotes task-shifting from nurses, who write prescriptions during routine ART clinic visits, to lay workers staffing the external CCMDD pick-up points.

The most expensive input to intervention implementation was the time of clinic staff. For this reason, relatively simple but human resource-intensive interventions, such as ensuring the regular opening of windows and doors or implementing a queuing system that allows for coughing patients to be rapidly triaged and for other patients to wait in a sheltered area outdoors, were found to be more expensive than those interventions relying on capital investments and technology, such as installing ultraviolet germicidal irradiation (UVGI) systems.

Enablers identified ranged from relatively inexpensive capital investments, such as electric heaters to ensure thermal comfort in winter when windows are kept open, to more costly enhancements to training programmes for clinic staff and consultations with community representatives, to ensure lasting changes in work culture and local ownership of the interventions. Overall, the proportion of total intervention costs represented by the enablers was inversely proportional to the size of capital investment required by the intervention; and directly proportional to the intervention’s reliance on changes in the behaviour of patients and/or staff (Table 1). Correspondingly, the share of total costs represented by the enablers ranged from 0 for the retrofitting of buildings to 54% for introducing appointment systems and 100% for expanding the use of community sites for ART collection.

## Discussion

This analysis applied group model building, an established SDM approach, to elicit information on the health system constraints that operate in TB IPC in South Africa. This information was then used in a novel way to build a model of the local incremental costs of a set of TB IPC interventions implemented at two clinics in KwaZulu-Natal. The interventions were designed bearing in mind potential barriers to implementation and necessary investments to overcome these; then costed iteratively, based on feedback given by SDM participants. The resulting unit costs thus reflect information linking the implementation process to outcomes, and are a closer representation of the full opportunity costs of these interventions compared to those generated with standard costing methods, as they include the costs of relaxing health system constraints.

Despite the addition of enabling costs, the TB IPC interventions considered are substantially less expensive than other interventions for preventing TB transmission currently included in the South African National Strategic Plan for HIV and TB as well as in the Investment Case for TB, such as improving the timeliness and yield of facility-based TB screening by using more sensitive algorithms and contact tracing (National Department of Health and South Africa National AIDS Council, 2016, South Africa National Aids Council, 2017). Intensified facility-based TB case-finding was found to be the most effective intervention at reducing TB incidence in model-based analyses, but it is also extremely costly in the short- and medium-term, as it generates an increase in diagnosis and treatment costs further along the TB care cascade (Menzies *et al.*, 2016). In addition, its feasibility was found to be low in an empirical proof of concept analysis quantifying the constraints around TB diagnosis and treatment in South Africa, and the costs of relaxing these constraints were substantial (Bozzani *et al.*, 2018). If proved to be at least as effective as the measures currently funded, TB IPC interventions could shift the balance of resource allocation within the South African TB programme.

Further cost savings could be realized by considering the proposed TB IPC interventions as a package, thus allowing the costs of those enablers that are shared by more than one intervention to be spread across them. An example would be the costs of enhancing routine staff training and supervision, which are shared by all the interventions analysed with the exception of building retrofits. Similarly, gains in efficiency could be realized from scaling up the interventions to the regional and/or national level (Gomez *et al.*, 2020). In this application, the SDM approach was used to identify intervention designs that build on current practice uniquely specific to the two study clinics. SDM could in principle be used to assist with designing more universally scalable interventions. However, there is substantial variation in the implementation of TB IPC measures across provinces and regions in South Africa. This made it difficult to estimate the national level costs of such context-specific interventions as retrofitting buildings or establishing appointments and queuing systems, all of which are dependent on clinic characteristics and on processes that were not uniformly established in the past and are currently used with varying rates of success (Voce, 2020; Zwama *et al.*, 2020).

Another potential limitation of applying the SDM approach to a costing exercise is that its focus on the broader health system characteristics and pathways of action may lead to the identification of certain constraints that cannot be relaxed through financial investments (e.g. lifting the moratorium on UVGI) or that are otherwise ‘uncostable’. This may be because the interventions and enablers consist of novel activities for which sources of price and quantity data cannot be readily identified, such as setting up new CCMDD pick up points; or they may consist of high-level activities, such as redesigning training formats and materials based on data collected from routine monitoring and evaluation, the costs of which are above-site and difficult to allocate to specific interventions; or they may consist of activities that fall outside the remit of the health sector, such as improving public transport links to health facilities to support the implementation of a clinic appointment system that spaces patient visits throughout the day. While activities that do not incur a cost and those that fall outside the health sector might be excluded from an economic evaluation (depending on the perspective taken), additional data collection is needed for costing novel activities and for allocating and scaling the costs of shared above-site enablers, e.g. from pilot/demonstration projects or feasibility studies.

## Conclusions

SDM-informed costing allows for a comprehensive view of the health system influences on intervention impact and feasibility, in a way that may be superior to other, less participatory stakeholder consultation methods. By providing several occasions for interaction between different TB IPC stakeholders at the national and decentralized level, as well as between stakeholders and researchers, the group model building exercise presented in this analysis enabled a thorough process for validating and refining costing assumptions, including on the details of intervention and enabler design. For successful application of this approach in economic evaluation, further research is needed into ways of integrating insights from SDM, that are well suited to identifying above-service level costs, with more traditional costing methods, which usually focus more on service-level inputs. Such a combination can, e.g. smooth the process of linking costs into transmission model outputs, which are usually service-level units, as well as potentially inform the choice of a functional form for modelling costs at scale. SDM can also be useful for identifying intervention types that might not be cost-effective based on the share of total costs represented by the required enablers. Further analyses of interventions that are more or less reliant on capital investments or behaviour change, such as new preventive or diagnostic technologies, are needed to fully assess its potential applications in economic evaluation and priority setting.

## Supplementary Material

czab155_Supp

## Data Availability

The data underlying this article are available in the article and in its online supplementary material.
