# Correction to: Using system dynamics modelling to estimate the costs of relaxing health system constraints: a case study of tuberculosis prevention and control interventions in South Africa

**DOI:** 10.1093/heapol/czac110

**Published:** 2023-01-03

This is a correction to: Fiammetta M Bozzani, Karin Diaconu, Gabriela B Gomez, Aaron S Karat, Karina Kielmann, Alison D Grant, Anna Vassall, Using system dynamics modelling to estimate the costs of relaxing health system constraints: a case study of tuberculosis prevention and control interventions in South Africa, *Health Policy and Planning*, Volume 37, Issue 3, March 2022, Pages 369–375, https://doi.org/10.1093/heapol/czab155

In the originally published version of this manuscript, the incorrect supplementary material was included. The correct supplementary file is linked here, and has been corrected in the original paper online.

## Supplementary Material

czac110_Supp

