# Supplementary material for: Using system dynamics modelling to estimate the costs of relaxing health system constraints: a case study of tuberculosis prevention and control interventions in South Africa
Source: Health Policy Plan. 2021 Dec 24;37(3):369–75. doi: 10.1093/heapol/czab155 (PMC8896337; doi:10.1093/heapol/czab155)
Supplement: czab155_Supp [file czab155_supp.zip › Supplementary file v2 clean.docx]

**SUPPLEMENTARY MATERIAL**

**Using system dynamics modelling to estimate the costs of relaxing health system constraints: a case study of tuberculosis infection prevention and control interventions in South African health facilities**

Contents

[1. Model of the TB IPC system and interventions design 1](#_Toc89174642)

[1.1 Model development 1](#_Toc89174643)

[1.2 Description of core dynamics 1](#_Toc89174644)

[2. Price assumptions 4](#_Toc89174645)

[3. Quantities and other cost calculation parameter assumptions 6](#_Toc89174646)

# 1. Model of the TB IPC system and interventions design

## 1.1 Model development

Figure S1 presents an overview of the dynamics at play in the TB IPC system in South Africa, as described by group model building workshop participants and subsequently synthesised using researchers’ notes, monthly check-in phone calls with participants and a second round of workshops to verify model structure and assumptions. The full process of elicitation and causal loop diagram development is described in detail by Diaconu and colleagues (2021)^[[1]](#footnote-1)^.

Briefly, the aims and activities of each workshop were targeted to the participant groups invited. The workshop with national-level stakeholders focussed on identifying distal and macro-level health system and policy influences on TB prevention and care. Activities with the practice stakeholders focused on identifying proximal factors related to TB care delivery and nosocomial transmission. On both days, participants were invited to reflect on key events and factors influencing changes in the TB burden over time. Participants were prompted to reflect on how the changing burden related to changes in TB and HIV care.

Variable elicitation exercises then followed, whereby participants could identify and hone in on individual variables which corresponded to either distal or proximal influences on TB transmission at primary care levels. Drawing on these variables, an experienced SDM facilitator and modeller guided participants in the development of causal loop diagrams depicting how policy influences primary care-level practices, which in turn affect *Mtb* transmission at clinic level. Areas of the system which were amenable to change, and/or which depicted issues of particular concern, were denoted as ‘points of fragility’. Focusing on these areas, participants were then asked to free-list intervention mechanisms that could address nosocomial transmission. Finally, these interventions were ranked by participants based on their perceived feasibility and impact. Their underliying mechanisms of action were further refined during monthly check-in calls.

## 1.2 Description of core dynamics

Figure describes areas of fragility within the system (in red), areas where intervention would be feasible (in blue) and areas that are identified both as fragile and also as potential candidates for immediate intervention (in purple). There are three core dynamics in the model.

The first one, in the bottom left corner, refers to *Mtb* transmission (T) in primary care clinics and how it relates to long waiting times and crowding (W&C). Both of the feedback loops depicted in this corner are reinforcing. For the transmission loop, this means that as the number of persons coming to the clinic increases at specific times, the likelihood of bottlenecks in patient flow occuring also increases. As more persons crowd in the same space, the probability of transmission of *Mtb* and the number of persons infected, in turn, also increase over the long term (the time delay between events is indicated via the broken arrow from ‘Number of people infected’ to ‘Number of people using the clinic’).

While this first loop mainly describes patient behaviours, it is important to realize that these behaviours are shaped by clinic processes and dynamics. Workshop participants also detailed how bottlenecks in patient flow occur due to ineffective administrative and clinical processes; this means that, in practice, more patients will crowd at specific places as they wait for services and, over time, the waiting times for seeing specific clinicians within the facility also increases and in turn may drive the occurrence of more bottlenecks.

**Figure S1. Causal loop diagram of TB IPC system**

The effectiveness of clinical and administrative processes is further affected by clinic culture, depicted on the right. This second dynamic refers to the existence of a ‘culture of compliance’ (CoC), whereby staff adhere to IPC interventions passively, by virtue of conformity rather than active engagement. The loop here describes how this, in turn, causes low morale and issues with staff retention (Ret loop).

Both the transmission and clinic culture dynamics are affected by a third dynamic related to system learning, at the top of the diagram. This relates to the overarching policy dynamics that ultimately shape clinic environment.

Interventions to address the problem areas, and the complementary enablers, were in the first instance suggested by participants themselves based on their own knowledge and experience. This information was then supplemented by the workshop facilitators based on the current TB IPC Guidelines published by the World Health Organization^[[2]](#footnote-2)^, the South African IPC framework^[[3]](#footnote-3)^ and manual^[[4]](#footnote-4)^, the Ideal Clinics Initiative checklist^[[5]](#footnote-5)^ and a scoping review of the literature on the barriers to implementing TB IPC interventions conducted by *Umoya omuhle* researchers^[[6]](#footnote-6)^.

# 2. Price assumptions

Ingredients prices used in the estimation of unit costs are summarised in Table S1.

**Table S1. Price data summary**

|  | **Ingredient** | **Unit price (2019 US$)** | **Unit** | **Source** |
| --- | --- | --- | --- | --- |
| **Intervention specific costs** |  |  |  |  |
| Staff time | Nurse minute | 0.36 | per minute | (1) |
|  | Outpatient visit - Nurse Cost | 1.45 | per visit | (1) |
|  | Outpatient visit - Other Cost | 4.32 | per visit | (1) |
|  | General worker minute | 0.03 | per minute | (1) |
|  | Administrator/data clerk minute | 0.10 | per minute | (1) |
| Building retrofits | Lattice brickwork installation | 187 | 1x1.5 m | (2) |
|  | Raising roof | 1,000 | per item | (3) |
|  | Turbine ventilators installation | 350 | per item | (4) |
| Personal protective equipment | N95 respirator | 2.50 | per item | (5) |
|  | N95 respirator fit testing | 6.12 | per event | (6) |
|  | Surgical mask | 0.37 | per item | South African retail price |
| UVGI | Clinic 1 UV lamps and installation | 47,672 | per event | Local supplier quote |
|  | Clinic 2 UV lamps and installation | 22,677 | per event | Local supplier quote |
|  | Life-cycle maintenance | 766 | per unit | (7) |
|  | Air mixing system | 1,500 | per clinic | (7) |
|  | Acceptance testing | 75 | per clinic | (7) |
|  | GUV meter | 2,000 | per clinic | (7) |
| ART | Antiretroviral therapy | 60 | per patient month | (8) |
| **Enabler costs** |  |  |  |  |
| Training and public health messaging | one-day stand-alone nurse training | 372 | per person | Local supplier quote |
|  | one-day stand-alone lay worker training | 102 | per person | Local supplier quote |
|  | one-day add-on nurse training | 223 | per person | (9) |
|  | one-day add-on lay worker training | 69 | per person | (9) |
|  | IEC materials/job aids | 0.18 | per person | (9) |
| Thermal comfort | Electric heater | 51 | per unit | South African retail price |
|  | Electric fan | 20 | per unit | South African retail price |

ART: Antiretroviral Therapy. IEC: Information, Education and Communications materials. UVGI: Ultraviolet Germicidal Irradiation.

(1) Bozzani FM, Mudzengi D, Sumner T, Gomez GB, Hippner P, Cardenas V, et al. Empirical estimation of resource constraints for use in model-based economic evaluation: an example of TB services in South Africa. Cost effectiveness and resource allocation: C/E. 2018;16:27.

(2) Taylor JG, Yates TA, Mthethwa M, Tanser F, Abubakar I, Altamirano H. Measuring ventilation and modelling M. tuberculosis transmission in indoor congregate settings, rural KwaZulu-Natal. Int J Tuberc Lung Dis. 2016;20(9):1155-61.

(3) Escombe AR, Ticona E, Chavez-Perez V, Espinoza M, Moore DAJ. Improving natural ventilation in hospital waiting and consulting rooms to reduce nosocomial tuberculosis transmission risk in a low resource setting. BMC Infect Dis. 2019;19(1):88.

(4) Cox H, Escombe R, McDermid C, Mtshemla Y, Spelman T, Azevedo V, et al. Wind-driven roof turbines: a novel way to improve ventilation for TB infection control in health facilities. PLoS One. 2012;7(1):e29589.

(5) End Tuberculosis Transmission Initiative (ETTI). Technical information sheet. Personal respiratory protection. Available from: <http://www.stoptb.org/wg/ett/assets/documents/ETTI_InfoSheet_Respirators_Final.pdf>

(6) End Tuberculosis Transmission Initiative (ETTI). Technical information sheet. Respirator fit testing. Available from: <http://www.stoptb.org/wg/ett/assets/documents/ETTI_InfoSheet_FitTesting_Final.pdf>

(7) End Tuberculosis Transmission Initiative (ETTI). Technical information sheet. Disinfecting room air with upper-room (UR) germicidal UV (GUV) systems. Available from: <http://www.stoptb.org/wg/ett/assets/documents/ETTI_TechSheet_GUV_final%20version.pdf>.

(8) Tagar E, Sundaram M, Condliffe K, Matatiyo B, Chimbwandira F, Chilima B, et al., Multi-country analysis of treatment costs for HIV/AIDS (MATCH): Facility-level ART unit cost analysis in Ethiopia, Malawi, South Africa and Zambia. PloS ONE. 2014;9(11)

(9) Jamieson L, Gomez GB, Rebe K, Brown B, Subedar H, Jenkins S, et al. The impact of self-selection based on HIV risk on the cost-effectiveness of preexposure prophylaxis in South Africa. AIDS. 2020;34(6):883-91.

# 3. Quantities and other cost calculation parameter assumptions

Annual ingredients quantities and other assumptions made in the calculation of interventions and enablers unit costs are summarised in Tables S2 and S3, respectively.

**Table S2. Annual quantities and other intervention parameter assumptions**

| **Interventions** | **Ingredients** | **Quantity** | | **Assumptions** |
| --- | --- | --- | --- | --- |
|  |  | **Clinic 1** | **Clinic 2** |  |
| 1. Improving ventilation by opening windows and doors | Nurse minutes | 40,320 | 16,128 | One clinical member of staff doing a round of the clinic every working hour (n=8) for every working day (n=252). Rounds take 20 minutes per hour at Clinic 1 and 8 minutes per hour at Clinic 2. |
| 2. Building retrofits | Lattice brickwork, 1x1.5m unit | 8 | 3 | Clinic 1: lattice brickwork along 1/5 of main corridor length and 1/7 of secondary corridor. Raise roof of secondary waiting area. Install 14 turbine ventilators in park homes. Clinic 2: lattice brickwork along 2/3 of width and 1/15 of length of main waiting area. Raise roof of main waiting area. Install 4 turbine ventilators in central area of main building |
|  | Roof raising | 1 | 1 |  |
|  | Turbine ventilators | 14 | 4 |  |
| 3. UVGI | GUV R30 | 6 | 2 | Clinic 1: 25 UVGI lamps in main building, 31 lamps in chronics building. Clinic 2: 26 UVGI lamps in clinic building. Count based on assessment by local supplier |
|  | GUV R31 | 14 | 10 |  |
|  | GUV R32 | 36 | 14 |  |
| 4. Surgical mask wearing for patients and N95 respirators for staff | N95 respirators | 2,608 | 782 | One N95 respirator per clinical member of staff every 5 shifts (50% coverage), fit-tested annually. One surgical mask per patient per visit (70% coverage) |
|  | N95 fit testing | 103 | 32 |  |
|  | Surgical masks | 10,749 | 5,374 |  |
| 5. Maximising use of existing CCMDD facilities | ART nurse visits per year, ineligible patients | 12 | 12 | CCMDD intervention ensures all stable patients (92%) receive 6-months repeat prescriptions so nurse visits reduced to two per year. Ten remaining visits occur at CCMDD point staffed by lay worker. From Umoya omuhle social contacts survey data, it is estimated that maximising use of the CCMDD system will lead to a 31% reduction in clinic visits costs (1). |
|  | ART nurse visits, eligible patients | 2 | 2 |  |
|  | ART lay worker visit | 10 | 10 |  |
| 6. Queue management system | General worker minutes | 7,678 | 3,839 | One designated nurses triaging coughing patients and one lay queue marshal directing the queue at each clinic (half a minute per visit). |
|  | Nurse minutes | 7,678 | 3,839 |  |
| 7. Appointments system | Administrator/data clerk minutes | 45,360 | 30,240 | One extra hour per day for clerk to pre-retrieve files and record appointments (two clerks at Clinic1). One hour for public awareness messaging in waiting area |

ART: Antiretroviral Therapy. CCMDD: Central Chronic Medicines Dispensing and Distribution. UVGI: Ultraviolet Germicidal Irradiation

(1) McCreesh N, Karat AS, Baisley K, Diaconu K, Bozzani F, Beckwith P, Yates T, Deol A, White RG, Grant A. Effect of infection prevention and control measures on rate of *Mycobacterium tuberculosis* transmission in primary health clinics in South Africa. Forthcoming

**Table S3. Annual quantities and other enabler parameter assumptions**

| **Enablers** | **Interventions enabled** | **Quantity** | | **Assumptions** |
| --- | --- | --- | --- | --- |
|  |  | **Clinic 1** | **Clinic 2** |  |
| Electric heaters | Opening windows and doors | 7 | 3 | Heaters positioned in waiting areas to ensure thermal comfort |
| Electric fans | Opening windows and doors | 30 | 8 | Fans positioned in consultation rooms to ensure thermal comfort |
| Onsite stand-alone staff training, people trained | UVGI | 45 | 14 | One day training for each intervention, repeated every 3 years |
|  | Opening windows and doors |  |  |  |
|  | N95 for staff and surgical masks for patients |  |  |  |
| Onsite add-on staff training, people trained | Appointments system | 45 | 14 | Half-day add-on training for each intervention, delivered in combination with other routine training, repeated every 3 years |
|  | Queuing system |  |  |  |
|  | Maximising use of CCMDD |  |  |  |
| Increased supervision from District Health Manager, days | Opening windows and doors | 54 | 54 | At introduction: daily facility visits for one month, weekly for another 2 months  Post-introduction: monthly visits |
|  | Appointments system |  |  |  |
| Offsite community workshops | Appointments system | 120 | 120 | Four sessions with 30 attendees each, once-off. Equivalent to stand-alone lay worker training |
|  | Queuing system |  |  |  |
|  | Maximising use of CCMDD |  |  |  |
| Information, education and communication materials | N95 for staff and surgical masks for patients | 1,536 | 768 | Information leaflets on surgical mask use disseminated around clinic for one in every ten patients |

CCMDD: Central Chronic Medicines Dispensing and Distribution. UVGI: Ultraviolet Germicidal Irradiation

1. Diaconu, K., Karat, A., Bozzani, F., Falconer, J., McCreesh, N., Voce, A., Vassall, A., Grant, A. & Kielmann, K. 2021. Using system dynamics modelling to support implementation of tuberculosis infection prevention and control measures in South African primary care facilities. *Under review*. [↑](#footnote-ref-1)
2. World Health Organization. WHO Guidelines on tuberculosis infection prevention and control, 2019 update. Geneva: World Health Organisation; 2019. [↑](#footnote-ref-2)
3. South Africa National Department of Health. National Infection Prevention and Control Strategic Framework. Pretoria: NDOH; March 2020. [↑](#footnote-ref-3)
4. South Africa National Department of Health. Practical Manual for Implementation of the National Infection Prevention and Control Strategic Framework. Pretoria: NDOH; March 2020. [↑](#footnote-ref-4)
5. South Africa National Department of Health. Ideal Clinic Framework - version 19. Pretoria: NDOH; May 2021. [↑](#footnote-ref-5)
6. Zwama G, Diaconu K, Voce AS, O'May F, Grant AD, Kielmann K. Health system influences on the implementation of tuberculosis infection prevention and control at health facilities in low-income and middle-income countries: a scoping review. BMJ Glob Health. 2021;6:e004735 [↑](#footnote-ref-6)
